# Evaluation of the Removal of Indicator Bacteria from Domestic Sludge Processed by Autothermal Thermophilic Aerobic Digestion (ATAD)

**DOI:** 10.3390/ijerph7093422

**Published:** 2010-09-02

**Authors:** Anna V. Piterina, John Bartlett, Tony J. Pembroke

**Affiliations:** 1 Department of Chemical and Environmental Sciences and Material and Surface Science Institute, University of Limerick, National Technological Park, Castletroy, Limerick, Ireland; E-Mail: anna.piterina@ul.ie (A.P.); 2 Sligo Institute of Technology, Cente for Sustainability, Sligo, Ireland; E-Mail: bartlett.john@itsligo.ie (J.B.)

**Keywords:** domestic sludge, safety, pathogen detection and inactivation, ATAD treatment efficiency, mobile element inactivation, SXT/R391, DN*ases*

## Abstract

The degradation of sludge solids in an insulated reactor during Autothermal Thermophilic Aerobic Digestion (ATAD) processing results in auto-heating, thermal treatment and total solids reduction, however, the ability to eliminate pathogenic organisms has not been analysed under large scale process conditions. We evaluated the ATAD process over a period of one year in a two stage, full scale Irish ATAD plant established in Killarney and treating mixed primary and secondary sludge, by examining the sludge microbiologically at various stages during and following ATAD processing to determine its ability to eliminate indicator organisms. *Salmonella* spp. (pathogen) and fecal-coliform (indicator) densities were well below the limits used to validate class A biosolids in the final product. Enteric pathogens present at inlet were deactivated during the ATAD process and were not detected in the final product using both traditional microbial culture and molecular phylogenetic techniques. A high DN*ase* activity was detected in the bulk sludge during the thermophilic digestion stage which may be responsible for the rapid turn over of DNA from lysed cells and the removal of mobile DNA. These results offer assurance for the safe use of ATAD sludge as a soil supplement following processing.

## 1. Introduction

From a public health perspective [1] the need for effective waste treatments and sludge stabilisation is ever increasing. With increased travel [2] and tourism the potential for diffusion of infectious diseases has increased and throughout Europe and elsewhere many diseases have (re)-emerged resulting in major health, ecological, socio-economical and political consequences. The establishment of EDEN (Emerging Diseases in a changing European Environment; http://www.eden-fp6project.net) is attempting to spread the message of the importance of developing and implementing advanced wastewater and sludge treatment systems [3]. As an alternative to standard sludge treatment systems, a new, energy-efficient, non-chemical process, autothermal thermophilic aerobic digestion (ATAD) was introduced over the past decade or so [4]. ATAD is a biological sludge treatment process that utilises the aerobic degradative abilities of microorganisms to convert soluble organic materials to lower energy forms [5]. Microbial catabolic processes resulting from aerobic oxidation of biosolids, gives rise to significant heat generation, which in turn results in thermal processing of the sludge [6–10]. Typically ATAD processes that operate to treat domestic sludge, following primary and secondary wastewater processing, involve sludge thickening, followed by a two stage thermal treatment where the sludge is highly aerated and the sludge temperature allowed to rise naturally due to microbial metabolism in insulated reactors [4,6,8]. This metabolic activity reduces the sludge solids content while the elevated temperatures should effectively stabilise the sludge and remove pathogens. In typical ATAD systems treating domestic sludge the temperature ranges between 45 °C and 65 °C, which ensures the best degradation rates while the temperature rise aids pathogen reduction. However above 60 °C, the degree of microbial diversity is markedly decreased, at least in ATAD systems treating pharmaceutical wastes, with negative consequences on the degradation process [11].

Biosolids reuse and disposal practices are currently regulated in Ireland by EU directive [12] and by a code of practice for biosolids reuse [13].The EU directive suggests a treatment regime of 55 °C for 20 hours, an absence of *Salmonella* in 50 g of sludge and process conditions to reduce *E. coli* CFU to <500 g^−1^. The pasteurisation ability of an ATAD process to produce such a class A biosolids is determined by the interplay between operational parameters, feed composition and its biodegradability, the diversity of the ATAD microbial consortia and their metabolic capacities and reactor design features such as insulation and aeration control [9,14] and thermotolerent coliforms and *Enterococci* are most often used as indicator organisms to assess the hygienic quality of such treated organic waste [15–23]. In addition *Salmonella* is a relatively common excreted pathogen, often present in sludge and sewage and hence is also monitored as an indicator organism [17,22,24].

The effect of heat on microorganisms has mainly been studied with respect to pathogen removal in food and wastewater and it has been assumed that the death kinetics of enteric viruses and protozoa are similar [25]. The exponential law of disinfection, referred to as “Chick’s Law” relates the survival of pathogens and other microorganisms as a function of temperature:

$$X_{t}/X_{o}=e^{-k(t-to)}$$

where ***X***_***t***_ is the surviving fraction following treatment, ***X***_***o***_ is the starting population number, ***t-to*** is the treatment time interval, ***k*** is the specific decay rate [17,25,26]. In general, studies on the thermal effects and heat inactivation of pathogens have been carried out under laboratory conditions with application of test bacteria [17,27], with *Salmonella* and *Escherichia* spp. being used as model indicator bacteria [28–30]. For the pathogen *Salmonella enterica*, inactivation is dependent on the strain type, and it is most effectively inactivated at 71 °C during 1.2 seconds [27] in a nutrient-rich environment. However, inactivation of enterococci in pure culture [17] required 40 days at 45 °C, 3 days at 50 °C, 15 hours at 55 °C, 2 hours at 60 °C, and 7 minutes at 70 °C. For pathogen inactivation in sludge [20], it has been shown that holding for 4 hours at 55 °C or for 30 minutes at 70 °C is effective in killing most pathogens, with killing depending on the matrix or media. The heat resistance of a pathogen is highly influenced by the strain tested, the type of experimental method used, culture conditions prior to the experiment and the heating method and the recovery conditions utilised. Thus there are many features that could add to variability in pathogen recovery under real sludge treatment conditions.

For sludge, adequate pathogen reduction can be obtained at time-temperature combinations that are described in detail by U.S. EPA’s time–temperature equation [5]. The time-temperature equation in the regulation requires that all particles be treated for a specified time at the temperature of operation. In brief, this requirement suggests a temperature and time of sludge treatment to achieve desirable quality of 65 °C for 1 hour, 75 °C for 136 seconds, 85 °C for 5.5 seconds or 55 °C for 24 hours. These requirements differ from the conditions necessary for pathogen inactivation in pure culture under laboratory conditions [25,30,32], as in wastewater or sludge microorganisms are often found within organic substances or embedded in flocs, which can protect them from pasteurization [25]. It has been demonstrated that treating domestic sludge at 60 °C for 35 minutes would reduce pathogens to acceptable levels [33], in comparison to pasteurization at 70 °C with a retention time of 30 min while at 60 °C full inactivation of pathogens within sludge requires a holding time of 4.78 h [5,34].

Cell-free nucleic acid is a potentially important source of energy and nutrients in sludge ecosystems [35,36]. However, little is known about the identity, metabolism, and interactions of the microorganisms capable of consuming cell-free DNA originating from cell lysis or from the original fecal materials. Some may undergo genetic transformation [37], some may be used for DNA or RNA synthesis [35,38] or it may give a valuable source of phosphorus depending on the ability of microorganisms to take up this macromolecule with passive transport limited to >0.6 kDa [39,40]. Some microorganisms are capable of degrading cell-free DNA with extracellular nucleases [42] and then consume the hydrolyzed products [35], while DNA that interacts with sludge material may be protected [42]. DNA hydrolysing bacteria from aquatic environments have been described [43,44] and assigned to mesophilic or psychroplilic organisms in the main. In wastewater ecosystems, it can be expected that the size and availability of the cell-free DNA and how microorganisms interact with it will have a strong effect on the diversity and metabolism of micro-organisms in wastewater and sludge [45]. There have been few reports on such activities with only one by Ruiz [46] describing the detection of DN*ase* activity in mesophilic anaerobic wastewater sludge. No data on DN*ase* production by thermophilic organisms and its possible role in thermal treatment of sludge has been reported. Such DN*ases* may also play a key role in preventing transmission of viruses, or mobile elements from sludge to the wider environment via gene transfer [47] which has contributed to genome plasticity and dissemination of fitness-enhancing traits, including antibiotic resistance and virulence factors [48–50]. Recent studies suggest that sludge is a specific location where genetic exchange can occur [47] and limiting such exchange may be particularly important for domestic sludge where antibiotic-resistant bacteria occur [51–54]. Although lysis can occur, natural transformation and DNA uptake is known to be responsible for genetic spread under mesophilic treatment conditions [47,55–58] and may even occur at thermophilic processing temperatures [59].

The first full scale ATAD plant to operate in Ireland is located in Killarney to treat locally produced primary and secondary sludge [60]. Insufficient treatment could lead to contamination by pathogenic microorganisms when the stabilised sludge is utilised for land spread. Thus we wished to determine the suitability of this ATAD process and sludge type for pathogen reduction [21] by monitoring the sludge seasonally using traditional culture based and molecular profiling techniques. We also wished to examine the effect of high levels of nuclease activity detected in the sludge on eliminating mobile DNA elements responsible for transmission of antibiotic resistance determinants.

## 2. Experimental Section

### 2.1. ATAD Sludge Source and Sampling

ATAD sludge was sampled from a full scale ATAD plant treating mixed primary and secondary sludges at Killarney Ireland. The plant has been described previously [8–10,60]. Briefly, the ATAD process consists of sludge thickening to 4–6% Total Solids, concentration on a belt filter followed by aeration to allow degradation and thermal treatment. The daily feed rate for the Killarney ATAD is in the range of 15–30 m^3^ d^−1^. The thickened sludge undergoes thermophilic digestion in a two-reactor (Reactor 1A and 2A) semi-batch process before treated sludge is stored in holding tanks where the sludge goes anaerobic (when aeration ceases). Reactors 1A and 2A, of 110 m^3^ capacity, are operated in series with partially digested sludge being fed from reactor 1A with and operation temperature range of 35–49 °C to the second ATAD reactor termed 2A, where operation temperatures hold in the range 58–65 °C. The ATAD reactors are followed by one or two holding tanks (275 m^3^), where the sludge is cooled naturally. Details of the plant and operating parameters have been published [8,60]. Samples were taken aseptically at different stages of processing and from the middle of the reactors by a deep-water sampling device. Total solids (TS) was analysed as per Standard Method 2540 D [19].

### 2.2. Microbiological Analysis of ATAD Sludge Quality

#### 2.2.1. Enumeration and Detection of the Indicator Organisms

Fecal coliforms and total enterococci in feed sludge, thickened sludge and ATAD treated sludge samples were evaluated using a most probable number assay (MPN) according to method 1680 and 1681 [19]. Fermentation tubes were incubated for 48 hours at 35 °C and observed at both 24 and 48 hours for the presence of presumptive growth indicated by gas or acid production. Presumptive positive tubes were transferred to fermentation tubes containing sterile EC media and incubated at 44.5 °C for 24 hours. Results of the MPN procedure were reported in terms of MPN g^−1^ total solids calculated from the number of positive EC culture tubes. Positive control cultures were included in each assay.

Specific detection of *Salmonella* was carried out according to method 1682 [19]. Enrichment was performed in Selenite Brilliant Green Sulfite (SBG) broth followed by isolation on Xylose-Lysine Deoxycholate agar (XLD). XLD was also used for the isolation of *Shigella* spp. and *Providencia* spp. Culturable populations of indicator bacteria were enumerated by direct plating on appropriate selective media [19]. Additionally bacterial cells that might adhere to ATAD biosolids *in situ* were removed using a combination of homogenisation, detergents, and dispersants, to eliminate the potential of adhering coliforms in the system. The sludge was dissolved in 0.01 M sodium pyrophosphate containing 0.09% (v/v) Tween 80 and stirred at 150 rpm with a magnetic stirrer for 30 minutes or homogenised at 3,000 × g for periods up to 30 seconds or vortexed at high speed. After the disintegration procedure, a spread plate technique was used for isolation and quantification of organisms. Selective bacteriological media included Levine Eosin Methylene Blue Agar (EMB), MacConkey Agar (MAC) designed for microbiological examination of sewage were used [19]. All media was autoclaved at 121 °C for 15 minutes. Serial dilutions were carried out for plating on selective media at 35 °C for 24 hours for total coliforms, 35 °C for 48 hours for fecal streptococci and total aerobic colonies and at 44.5 °C for 24 hours for fecal coliforms.

#### 2.2.2. Biochemical Identification of Microorganisms

Bacterial appearance on selective agars following MPN selection or via direct plate counts were defined as typical or atypical for each bacterial species after 24 hours incubation and identified based on known characteristics [19,31,62–65] or deemed negative if none appeared. Bacterial isolates were identified via the API profiling system and via biochemical tests [66]. Colonies which showed atypical morphological appearance on selective media or which did not show reliable profiles during API identification were analysed via molecular profiling.

#### 2.2.3. Molecular Identification of ATAD Organisms via Intergenic Spacer Region Analysis

Molecular profiling of ATAD organisms involved PCR amplification of the bacterial intergenic spacer region between the genes encoding the small (16S) and large (23S) rRNA subunit in the bacterial rDNA operon, with oligonucleotide primers targeted to conserved regions between the 16S and 23S genes [68]. The 16S–23S intergenic region, which may encode tRNAs, depending on the bacterial species, displays significant heterogeneity in both length and nucleotide sequence [68] which has been extensively used to distinguish bacterial strains and closely related species [69,70]. Using the technique of Ribosomal Intergenic Spacer Analysis (RISA), the length heterogeneity of the intergenic spacer can be exploited. The PCR product (a mixture of fragments contributed by community members) was electrophoresed in a polyacrylamide gel, and the DNA visualized by silver staining. The resultant complex banding pattern provides an isolate-specific profile, with the PCR banding pattern corresponding to a specific bacterial species. In this study the technique was applied to maximise the number of screened bacterial isolates and to perform sufficient grouping between multiple species cultivated on selective media before further cloning and sequencings procedures were utilised. This approach was found to be rapid, useful and reliable in similar microbiological and diagnostics studies [71].

#### 2.2.4. DNA Extraction via the CTAB Extraction Method

Bacteria were cultured in TSB broth for 7 hours at 37 °C to an OD_600_ of 1.0. A 1 mL aliquot of culture was centrifuged at 4 °C for 20 minutes at 4,000 × g. Pellets were kept for DNA extraction and resuspended in 567 μL of TE buffer (10 mM Tris Hal, 1 mm EDTA, pH 7.5) by gentle mixing. 30 μL of 10% SDS and 3μL of 20 mg mL^−1^ proteinase K was added and incubated for 1 hour at 37 °C when 100 μL of 5 M NaCl solution was added. This was followed by addition of 80 μL CTAB/NaCl solution [72] and incubated for 10 minutes at 65 °C. Equal volumes of chloroform/isoamyl alcohol (24:1) were then added, mixed and centrifuged for 5 minutes at 13,000 × g. Supernatants were extracted with equal volumes of phenol/chloroform/isoamyl alcohol (15:24:1) mixed well and centrifuged for 5 minutes at 13,000 × g. 0.6 volumes of isopropanol were then added and the solution gently mixed before DNA precipitation by centrifugation at 13,000 × g for 15 minutes. The resultant pellet was washed twice with 0.5 mL of 70% ethanol followed by centrifugation at 13,000 × g for 5 minutes and the pellet air-dried for 1.5 hours. This was resuspended in 50μL of TE buffer and 1 μL of RN*ase* (DN*ase* free) (Roche) was added and incubated at 37 °C for 20 minutes to remove traces of the co-extracted RNA [72].

#### 2.2.5. RISA-PCR

RISA-PCR, reaction mixtures contained 1 × PCR buffer (Bio-Line), 3 mM MgCl_2_, 500 μg mL^−1^ of BSA, 200 μM of each dNTP, 400 μM of each primer, 2.5 U of *Taq* polymerase (BioLine) and approximately 2ng of template DNA in a final volume of 50 μL. The primers used were S926f (universal, 16S rDNA gene), and L189r (Table 1). Reaction mixtures were held at 95 °C for 5 minutes, followed by 30 cycles of amplification at 93 °C for 15 seconds, 53 °C for 1 minute, and 72 °C for 1.5 minutes and a final extension of 72 °C for 9 minutes. To investigate the effect of the PCR cycle number on RISA profiles, PCR was also performed with 15, 20, and 25 rounds of amplification by using samples of the bacterial DNA. Reaction volumes were 10, 20, and 50 μL, with reagent concentrations as described above, except for the template DNA, which was increased by 1.5 to 3 times the usual amount. 15 μL aliquots were separated by electrophoresis on a native 6% polyacrylamide gel run for 12 or 17 hours at 8mA, respectively. Gels were stained with SYBR green safe II (Molecular Probes, Leiden, The Netherlands).

#### 2.2.6. PCR Amplification of the V6-V8 Region of 16S rDNA Genes for Phylogenetic Identification

Primers U968f and L1401r (Table 1) were used to amplify a 402-bp section of the bacterial 16S rDNA gene, including the highly variable V6 region [67,74]. Specific amplification of the target sequences was routinely achieved using 1–10 ng of template DNA in a total volume of 80 μL PCR reaction mixture [300 mg mL^−1^ BSA, 1.25 nmol mL^−1^ of each primer, 200 mM of each dNTP, PCR buffer, and 5 U of *Taq* polymerase (Bio-Line)].

After an initial denaturation step of 3 min at 94 °C, PCR temperature cycles of 1 minute denaturation at 94 °C, 1 minute of annealing, and 1 minute of primer extension at 72 °C were performed. During 10 initial touchdown cycles, the annealing temperature was lowered from 56 °C to 47 °C in steps of 1 °C per cycle. Subsequently, 25 cycles of 46 °C followed by a final extension step of 10 minutes at 72 °C were carried out. PCR products were purified on 0.8% (w/v) agarose gels using the ‘Promega’ PCR Clean-Up kit. PCR products were separated by electrophoresis on a native 6% polyacrylamide gel run for 2 hours at 8mA. Gels were stained with EtBr and observed under UV light.

#### 2.2.7. Cloning of the 16S rDNA Amplicons

Purified PCR product was directly ligated into the pGEM-T vector cloning system (Promega) and transformed into competent cells as described by the manufacturer. Extracted plasmids were used as a template for DNA sequencing. DNA sequencing was performed using a modified version of the di-deoxy chain termination method [75], using the SequiTherm Excel II DNA sequencing KiT-LC (Epicentre Technologies, Madison, WI, USA), and fluorescence DNA primers (MWG-Biotech (Milton Keynes, London, UK), labelled at the 5’- end with the dye IRD-800 (Li-COR Inc., Lincoln, NE, USA). Partial 16S rDNA sequences were compared with those in publicly accessible databases by using the program Basic Local Alignment Search Tool (BLAST), at the NCBI [76].

### 2.3. Assessment of Sludge Bulk Water for Nuclease Activity with Potential to Degrade Cell-Free DNA

Five milliliters of sludge obtained from the thermophilic stage of the ATAD reactor was centrifuged at 3,000 × g. 200 ng of the genomic DNA extracted from *E .coli* JM109 was added to 1 μL of diluted sludge bulk water. Separate tubes were incubated at both 37 °C and at 55 °C for 1 hour and loaded onto a 0.9% agarose gel (TAE buffer). Changes in the integrity and molecular weight of the loaded DNA sample were recorded as an effect of nuclease activity [46].

### 2.4. SXT/R391-like ICE Mobile Element Detection

Inlet sludge and product sludge were collected and DNA extracted from the sludge as described. The product was additionally washed to remove all cell-free DNA in the bulk water. Following phenol-chloroform extraction the slurry was centrifuged at 13,000 × g and DNA precipitated from the aqueous phase by isopropanol. Primer pairs IntFor1 and IntRev1 (Table 1) capable of specific amplification of the hallmark integrase gene of SXT/R391-like enteric mobile genetic elements were utilised [73]. The PCR cycle for amplification of the integrase gene was 95 °C for 10 minutes (to denature the DNA), followed by 5 cycles of 95 °C for 45 seconds, 64.5 °C for 45 seconds, 72 °C for 1 minute, and 30 cycles of 95 °C for 45 seconds, 61.5 °C for 45 seconds, 72 °C for 1 minute, and a final elongation step of 72 °C for 7 minutes. The expected size of the amplicon was 1,378 bp [73]. Products of the PCR amplification were analysed on a 1% agarose TAE gel and recorded as negative or positive. Signal intensity and copy number estimation of the integrase gene was not analysed during this study.

## 3. Results

### 3.1. Alteration of Process Parameters during ATAD Treatment

**Figure 1.**
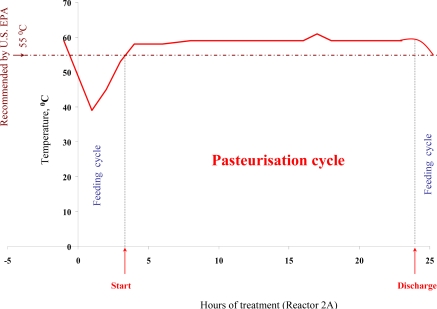
Characteristic operational temperature plot in reactor 2A of the feeding and pasteurisation stages during ATAD treatment at the Killarney ATAD.

Physico-chemical characteristics of sludge samples obtained from different stages of the ATAD process are summarized in Table 2. The overall digestion process was characterized by a continuous increase in the pH of the sludge from pH 6.3 in the feed to pH 9.1 in the thermophilic Reactor 2A. pH values have previously been described to correspond to the rate of biodegradation of the organic-rich material, particularly protein, during the processes, which is related to the release of ammonia. Due to the transformation of organic nitrogen to ammonia, and inhibition of microbial nitrification and denitrification at thermophilic temperatures [5], the gaseous ammonia is collected in the “gas-off scrubber” however some ammonia is solubilised and accumulates in the bulk sludge [4,6]. Because of this the pH changes gradually during the ATAD process to alkaline values as the temperature increases from 17 to 65 °C. While the temperature of reactor 1A remains at 43 °C, that of the second reactor 2A fluctuates due to the addition of new material from reactor 1A and the withdrawal of treated sludge to storage (Figure 1).

Temperature values in Reactor 2A of >56 °C are maintained for periods of 21 hours (shown as a pasteurisation cycle on Figure 1). Monitoring over many cycles demonstrated that this time-temperature regime was maintained during each pasteurisation cycle. The range of time-temperature cycles was found to fluctuate from 1,210–1,320 °C h^−1^ in the Killarney ATAD process which is higher than required by the EU directive for high quality sludge production [12].

This data indicated that the pasteurisation regime more than meets the EU regulations for thermal treatment of domestic sludge. The colour of the feed sludge underwent changes from grey to light brown during treatment in Reactor 1A (mesophilic-thermophilic stage) to dark brown after treatment in the thermophilic stage (Reactor 2A) and may be due to non-enzymatic browning of released sugars during prolonged thermal treatment. The total solids reduced from 6.3 to 4.2% during the ATAD treatment and this reduction was correlated to microbial activity induced temperature increase. The increase in total solids content (0.4%) during prolonged storage in the holding tank (Table 2, “Product 9 days”) may indicate a potential increase of viable biomass and re-growth of the some anaerobic mesophiles during this anaerobic mesophilic storage.

### 3.2. Microbiological Analysis of the Indicator Bacteria and Fate during the Sludge Processing

To examine the microbiological quality of the biosolids produced at the Killarney ATAD plant, sludge samples taken before and after ATAD treatment, and post-storage, were examined. After thickening of secondary treated sludge (to between 4.5 and 8.6% dry solids) the sludge is heated to approximately 35 °C and transferred to the ATAD reactor where it undergoes mixing and aeration until the target temperature of between 55 °C and 65 °C is attained. We implemented an optimal disintegration and dispersion method determined previously (*data not shown*) to separate and enumerate bacterial numbers from thermophilic sludge. To eliminate potential errors related to variability of cultivation-based methods on the recovery of indicator bacteria, several methods were applied in this study including most probable number [MPN] techniques and plating on various selective media.

#### 3.2.1. Non-Presumptive Bacterial Growth on the Selective Plates

The nature of the bacterial population present in ATAD is largely uncharacterised and therefore the nature of organisms that might grow on selective media (even atypical growth) needed to be characterized, especially following thermal treatments in ATAD. To identify these colonies we initially carried out Gram staining and then utilized the API system and some isolates were subjected to DNA extraction and 16S rDNA analysis by PCR amplification and sequence analysis [77,78]. In this study, we found that in almost all cases atypical growth on EMBA or MacConkey agar was due to growth of *Bacillus* species, closely related to *B. licheniformis* (with 95–100% similarity of the 16S rDNA sequences) while in the rest of the cases (<1%) growth was due to *Pseudomonas* species.

#### 3.2.2. Removal of Pathogens during ATAD Process

When specific tests for the presence of *Salmonella* sp. were carried out, following enrichment and selective plating, we identified the presence of *Salmonella* spp. in all the influent samples tested following secondary treatment. Confirmation of *Salmonella* was carried out using the API E20 *Enterobacteriaceae* test system and RISA molecular profiling. The data demonstrated that feed sludge supplied immediately after primary sludge treatment carried a higher density of indicator organisms (total and fecal coliforms) than in the feed sludge which underwent additional secondary treatment (Tables 3, 4, 5). However, these numbers were shown to be lower (4–7 logs) than reported for primary sludge in a similar study from the Czech Republic (9 logs) [15]. Following ATAD treatment we were unable to detect *Salmonella* on selective media either by direct plating, following enrichment or following pre-treatment of the sludge to release adhering organisms. Many of the indicator organisms which were abundant in the original feed were significantly reduced (up to 7 logs) after thermophilic digestion. Processed ATAD sludge was characterised by an absence of *Salmonella* spp. in biosolids samples, where up to 4 g of total solids were analysed at each stage. In control experiments, where select numbers of *Salmonella* were added to the effluent following treatment as a spike, these could be enumerated. This data indicated that the *Salmonella* sp. present in the effluent were in fact being inactivated by the thermal treatment and processing conditions rather than by any inhibitory effects of the effluent. This reduction in count upon treatment was equivalent to a minimum 5-*log* reduction in indicator organisms such as coliforms and *Salmonella* spp.

A key issue using such determinations is whether the lack of detection might be due to these indicator organisms being viable but stressed and non-culturable. Cell counts varied depending on selective media, while the count on MacConkey agar was comparatively higher compared to those obtained on EMB agar possibly due to sensitivity of the environmental isolates to the dyes in the selective media. These selective ingredients (e.g., EMB agar contains Eosin Y and Methylene Blue) may exert an adverse influence on the resuscitation of injured cells present in the sludge and alter their recovery differentially depending on the selective media and highlights just one of the disadvantages of utilising culture based methods. In fact it has been reported that pathogen re-growth may occur in sludges following storage [22–24]. At the Killarney ATAD plant the treated effluent is stored in large storage tanks for several months until it can be utilized for land spread. Over a 6 month period this stored ATAD sludge was sampled aseptically to determine the possibility of some form of thermal shock being responsible for our inability to culture such organisms from fresh thermophilic biosolids which could subsequently ‘resuscitate’. Treated ATAD sludge following prolonged storage within the holding tanks was therefore subjected to similar microbiological analysis by the same protocol as used to examine the non-treated sludge. Our data indicated that the thermal treatment appeared to kill the tested indicator organisms and that no re-emergence appeared to occur after post treatment steps under our experimental conditions.

#### 3.2.3. Seasonal Detection of Indicator Bacteria

Removal efficiency data for pathogens was consistent over the 1 year period sampled and was stable as a function of season (when load levels were high or low) and inlet microbial population fluctuations. In the summer, when the average human population contributing to the effluent is several times that in winter the amount of sludge undergoing treatment increases and of the numbers of indicator bacteria also increased. During this time of year (June–September) the ATAD system usually increased to 4 reactors with two operating in parallel, to increase the efficiency of the treatment without compromising the quality of final product. This approach has been operationally shown to be very effective in processing the increased quantity of sludge. Monitoring during both winter, when outside temperatures fall below freezing point, and summer periods revealed that the microbiological quality of the processed sludge (Table 5) met the regulations for Class A Biosolids for agricultural land application.

### 3.4. Exogenous DNA Degradation by the Sludge

**Figure 2.**
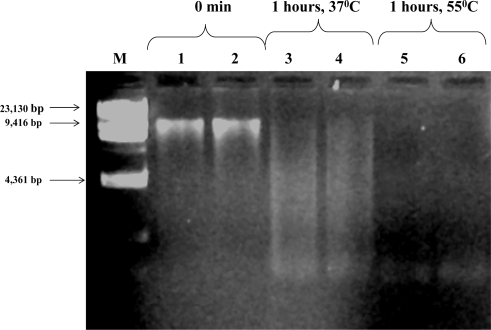
Exogenous DNA (extracted from *E. coli* JM109) degradation by liquid extracts from ATAD sludge incubated at different temperatures.

We observed that bulk water originating from the thermophilic ATAD sludge showed a high level of DN*ase* activity. We examined this by adding exogenous high molecular weight DNA (>13 kb) (Figure 2, Lanes 1 and 2) to ATAD derived liquid exudates and observed degradation in a short period of time during incubation at 37 °C, with low molecular weight products between 50–2,000 bp being obtained (Figure 2, Lanes 3 and 4). Incubating such mixtures of DNA and extract liquid at 55 °C, did not inhibit the degradation, but rather appeared to stimulate digestion (Figure 2, Lane 5 and 6). This observation was consistently associated only with the thermophilic stage of the treatment and liquid extracts from inlet or the low temperature reactor showed no equivalent degradation. It may be that as the temperature rises in the thermophilic reactor that lysis of mesophiles occurs releasing DNA and although they may release nucleases these may in fact be inactivated by the high temperature. Thus although such mesophiles may contribute DNA it is likely that the DN*ase* activities are associated with lysed thermophilic populations since the activity is not inhibited at the high temperature. We noted that the turnover rates for cell-free DNA were rapid and was approximately 30 min at 55 °C in vitro, suggesting that DNA may act as a high-quality nutrient capable of supporting extensive microbial metabolism at elevated temperatures. The presence of DN*ase* in the ATAD sludge was shown to be constant irrespective of season and may in fact be an intrinsic characteristic of the ATAD process particularly at the thermophilic stage (Table 6).

### 3.5. Fate of the Mobile Elements during the ATAD Treatment

We monitored the efficiency of removal of the SXT/R391 group of mobile elements or ICE’s (found to be present in inlet sludge) during the ATAD treatment, by utilising PCR probes (Table 6) specific to the unique ICE integrase gene as previously described [73]. SXT/R391 specific DNA was detected in inlet samples obtained at three periods of the year: during spring, summer and autumn, when there is an increase in tourist population numbers and when these elements may be imported from areas such as Asia where they are found regularly. During the winter sampling, however, the element was not detected. Samples taken from ATAD treated biosolids (from bulk sludge or particulate matter) however were PCR-negative for SXT/R391 elements at all times of the year indicating elimination of these mobile genetic elements. DN*ase* activity was detected during all seasons during sampling of ATAD wastewater.

## 4. Discussion

Although ATAD plants treating domestic sludge have been operating worldwide for many years, there has been limited systematic analysis of their actual potential to remove pathogens [14,15,79]. There has always been the ‘presumption’ that because of the time-temperature relationship between sludge holding in the thermophilic reactors that this would lead to the removal of coliforms, making the sludge pathogen free and allowing its classification as a Class A Biosolids.

There are, however, many factors that could effect this presumption. These range from inadequate holding times, thermal protection of organisms by the sludge biosolids [61] or re-growth of viable non-culturable coliforms after the sludge treatment process.

Bio-flocculation, production and embedding of microorganisms into a sludge Extra Cellular Polysaccharide (EPS) matrix, sludge structure, nutrient availability and heat distribution within reactors can all be important factors influencing the actual performance of a full-scale ATAD sludge treatment system. In addition, other factor such as climate, operation, and pathogen densities may fluctuate from site to site. Thus we have examined the thermal inactivation of pathogens in a full scale ATAD system at Killarney *de novo* over an extended 15 month period. Using a two-factor Weibull model based on temperature and retention time, we were able to obtain good agreement between calculated and observed rates of kill of *Salmonella* spp. and fecal coliforms (Figure 1). Times of exposure of the sludge to inactivation temperatures were found to be sufficient to meet the requirements of both the EU and the US EPA standards [5,12,13]. Our data provides evidence for pathogen reduction and also verifies their absence at the post treatment stage, during storage and prior to land spread. Autothermal processing capitalises on microbial exothermic reactions and process monitoring at the Killarney ATAD indicates that temperatures regularly reach 65 °C but can fluctuate depending on addition of new sludge and other process parameters [60].

In general ATAD temperatures operate between 55–65 °C on a consistent basis even allowing for addition of new sludge and removal of treated matter. In addition to heat, changes in pH (Table 2) due to the accumulation of NH_3_, the presence of metabolic antagonistic compounds produced by indigenous microflora, microbiological competition for nutrients with developing thermophilic populations [80,81] and water content may all play a synergistic role. During ATAD high levels of ammonia are released from the degradation of proteins and as its solubility increases with temperature, this result in pH changes in the bulk water. Ammonia is highly soluble in water which is partly explained by its polarity and ability to form hydrogen bonds [82]. In aqueous solution, ammonia acts as a weak base producing hydroxide ions by the de-protonation of water. Various authors have demonstrated that molecular ammonia has a bactericidal effect on enteric pathogen [83–86].

Culture based analysis of pathogens applied to ATAD sludge have limitations because of the possibility of bacteria entering a viable but non-culturable state (VBNC) as a strategy for temperature or adverse condition survival or the inability to provide growth conditions for unknown ATAD consortia. DNA based or microscopic analysis are thus needed for routine application which would provide data on the presence of pathogens, their survival during treatment and their potential out growth later during land application.

## 5. Conclusions

The Killarney ATAD was demonstrated to bring about effective pasteurisation and pathogen removal of municipal domestic sludge at scale and reduction of *Salmonella* spp. and microbial indicators of fecal contamination was demonstrated. Removal of indicator bacteria was consistent as a function of season over a 1 year test period (ranging between 5–7 logs). In addition to pathogen removal, the potential to remove mobile genetic elements was also observed. Our results support the hypothesis that a high nucleases activity observed in ATAD bulk water can play a beneficial role in reducing exogenous DNA (which could also potentially result in decreased viral loads) and highlights the need to examine such effects in other sludge treatment systems. Further research is needed to identify potential risks associated with cell-free DNA from sludge and its potential for transmission and persistence within soil to which such treated sludge is applied.

## Figures and Tables

**Table 1.** List of the oligonucleotides utilized in this study.

**Primer**	**Specificity**	**Sequence (5’-3’)**	**Position**	**T_h_^b^, (°C)**	***Refs.***
U968 a	Universal bacterial	AACGCGAAGAACCTTAC	968–984 nt, 16S rDNA	56	[67]
L1401		CGGTGTGTACAAGACCC	1,385–1,401nt,16S rDNA	56	[67]
S926f		CTYAAAKGAATTGACGG	910 to 926 nt, 16S rDNA	53	[70]
L189r		TACTGAGATGYTTMARTTC	189–207 nt, 23S rDNA	53	[70]
T7	pGEM-TA vector	TAATACGACTCACTATAGGG	T7 promoter	53	Promega,UK
SP6		ATTTAGGTGACACTATAG	SP6 promoter	53	Promega,UK
Int Forw	Integrase gene, R391 ICE element	AACTAGGGCTGGGCTTATAA CATGGCC	-------	56	[73]
Int Rev		AAAGATGGCAGCTTGCCGCA A CCTC	-------	56	[73]

a *E. coli* numbering;

b T_h_, Primer-specific annealing temperature

**Table 2.** Temperature, pH and Total Solids content of the sludge during the overall ATAD `process.

**Samples utilized in this study**	**Temp (°C)**	**pH**	**Total solid content (%)**

Feed (Pre thickened)	11	6.3	6.3
Reactor 1A (Stage 1)	43	7.0	5.8
Reactor 2A (2 h) (Stage 2 early)	53	8.1	5.1
Reactor2A (24 h)(Late Stage 2 prior to discharge)	63	9.1	4.2
Stabilised Product	14	7.8	4.6

**Table 3.** Microbiological quality of various sludges before and following treatment at the Killarney ATAD plant in relation to fecal and total coliform counts.

**Sample point**	**Fecal coliforms1(McConkey agar)**	**Total Coliforms (McConkey agar)**	**Fecal Coliforms (EMBA agar)**	**Total Coliforms (EMBA agar)**

Primary Sludge	3.6 × 10^5^	6.1 × 10^7^	2.1 × 10^4^	8 × 10^6^
Secondary Sludge	5.1 × 10^3^	7.1 × 10 ^5^	2 × 10^3^	3.5 × 10^4^
ATAD Product	<1	<1	<1	<1

1 The results presented are the average of 20 determinations. In all cases indicator organisms were not detectable in the ATAD product either on MacConkey and EMB selective agars. Data presented are cfu g^−1^ of dry sludge.

**Table 4.** Microbiological quality of various sludges before and following treatment at the Killarney ATAD plant in relation to fecal and total coliforms, *Salmonella* spp. and enterococci counts.

**Sampling point**	**Fecal Coliforms**	**Total Coliforms**	***Salmonella*spp.**	**Total Enterococci**

Primary Sludge	3.6 × 10^5^	6.1 × 10^7^	1.2 × 10^4^	7.3 × 10^5^
Secondary Sludge	5.1 × 10^3^	7.1 × 10 ^5^	8 × 10^2^	9 × 10^3^
ATAD Product	Non detectable < 1	Non detectable < 1	Non detectable < 1	Non detectable < 1

**Table 5.** Seasonal data for microbiological quality (fecal coliforms, enterococci, *Salmonella* spp.) for ATAD processed sludge assessed by the MPN method.

**Sample point**	**Fecal*Coliforms*(MPN/100 mL)**	**Total*enteroccoci*(MPN/100 mL)**	***Salmonella*sp.**

Product (March)	below detectable limit	below detectable limit	below detectable limit
Product (July)	below detectable limit	below detectable limit	below detectable limit
Product (October)	below detectable limit	below detectable limit	below detectable limit
Product (January)	below detectable limit	below detectable limit	below detectable limit
Detection limit	20 1	20	10

1 Detection limits in liquid as used in the MPN are higher than those used on solid media as shown in Tables 3 and 4.

**Table 6.** PCR analysis of the fate of SXT/R391-like ICE mobile genetic elements during ATAD treatment.

**Sampling Point**		**Season**			
		**Winter**	**Spring**	**Summer**	**Autumn**
		**January**	**March**	**July**	**October**
**Inlet**		*ND^1^*	*D*	*D*	*D*
**Biosolids**	Bulk water	*ND*	*ND*	*ND*	*ND*
	Particulate matter	*ND*	*ND*	*ND*	*ND*

1 SXT/R391-like ICE’s were detected using PCR and probes (IntFor1 and IntRev1) to the unique ICE integrase gene. (Table 1). Results were scored visually and recorded qualitatively as detected (D) or not (ND). Positive controls or R391 were utilised and samples spiked with R391 to show its removal.

## References

[1] Feachem RG Bradley DJ Garelick H Mara DD Sanitation and Disease: Health Aspects of Excreta and Wastewater Management John Wiley and Sons Chichester, UK 1983

[2] Miguéns JL Mendes JF Travel and tourism: into a complex network Physica A 2008 387 1

[3] Tchobanogluous G Burton FL Wastewater Engineering: Treatment, Disposal, and Reuse Irwin/McGraw-Hill Boston, MA, USA 1991

[4] Kelly HG Melcer H Mavinic DS Autothermal thermophilic aerobic digestion of municipal sludges: A one-year, full-scale demonstration project Water Environ. Res 1993 657 849 861

[5] Autothermal Thermophilic Aerobic Digestion of Municipal Wastewater Sludge Report EPA/625/10-90/007; US Environmental Protection Agency Washington, DC, USA 1990

[6] Kelly HG Emerging processes in biosolids treatment J. Environ. Eng. Sci 2006 5 175 186

[7] LaPara TM Alleman JE Thermophilic aerobic biological wastewater treatment Water Res 1999 33 895 908

[8] Piterina AV MacCusland C Bartlett J Pembroke JT Microbial ecology of auto-thermal aerobic digestion (ATAD): Diversity, dynamics and activity of bacterial communities involved in treatment of a municipal wastewater’ Recent Advances in Applied Microbiology; Understanding and Exploiting Microbes and Their Interactions Biological, Physical, Chemical and Engineering Aspects Formatex Badajos, Spain 2006 210 221

[9] Layden NM Mavinic DS Kelly HG Moles R Bartlett J Autothermal thermophilic aerobic digestion (ATAD)—Part I: Review of origins, design, and process operation J. Environ. Eng. Sci 2007 6 665 678

[10] Layden N Kelly H Mavinic D Moles R Bartlett J Autothermal thermophilic aerobic digestion (ATAD)—Part II: Review of research and full-scale operating experiences J. Environ. Eng. Sci 2007 6 679 690

[11] LaPara TM Nakatsu CH Pantea L Alleman JE Phylogenetic analysis of bacterial communities in mesophilic and thermophilic bioreactors treating pharmaceutical wastewater Appl. Environ. Microbiol 2000 66 3951 3959 10966414 10.1128/aem.66.9.3951-3959.2000 PMC92244

[12] EU Sludge Directive Available online: http://ec.europa.eu/environment/waste/sludge/index.htm (accessed on 1 August 2010).

[13] Codes for Good Practise Department of the Environment and Local Government & Department of Agriculture and Food and EPA Dublin, Ireland 1994 Available online: http://www.environ.ie/en/Publications/Environment/Water/FileDownLoad,17228,en.pdf (accessed on 1 August 2010).

[14] Ugwuanyi JO Harvey LM Mcneil B Effect of process temperature, pH and suspended solids content upon pasteurization of a model agricultural waste during thermophilic aerobic digestion J. Appl. Microbiol 1999 87 387 395 10540241 10.1046/j.1365-2672.1999.00831.x

[15] Zabranska J Dohanyos M Jenicek P Ruzicikova H Vranova A Efficiency of autothermal thermophilic aerobic digestion and thermophilic anaerobic digestion of municipal wastewater sludge in removing *Salmonella* spp. and indicator bacteria Water Sci. Technol 2003 47 151 156 12639021

[16] Watanabe H Inactivation of pathogenic bacteria under mesophilic and thermophilic conditions Water Sci. Tech 1997 36 25 32

[17] Strauch D Pathogenic micro-organisms in sludge. Anaerobic digestion and disinfection methods to make sludge usable as a fertiliser Eur. Water Manage 1998 1 12 26

[18] Ayres RM Mara DD Analysis of Wastewater for Use in Agriculture: A Laboratory Manual of Parasitological and Bacteriological Techniques WHO Geneva, Switzerland 1996

[19] Standard Methods for the Examination of Water and Wastewater19th ed American Public Health Association Washington, DC, USA 1995

[20] Carrington EG Davis RD Hall JE Pike EB Smith SR Unwin RJ Review of the scientific evidence relating to the controls on agricultural use of sewage sludge Report DETR4415/3 [part1] and Report DETR 4454/4 WRC Publications Medmenham, UK 1998

[21] Gerba CP Pepper IL Whitehead LF A risk assessment of emerging pathogens of concern in the land application of biosolids Water Sci. Technol 2002 46 225 230 12479475

[22] Sidhu J Gibbs RA Ho GE Unkovich I Selection of *Salmonella typhimurium* as an indicator for pathogen regrowth potential in composted biosolids Lett. Appl. Microbiol 1999 29 303 307 10664970 10.1046/j.1365-2672.1999.00626.x

[23] Zaleski KJ Josephson KL Gerba CP Pepper IL Potential regrowth and recolonization of salmonellae and indicators in biosolids and biosolid-amended soil Appl. Environ. Microbiol 2005 71 3701 8708 16000779 10.1128/AEM.71.7.3701-3708.2005 PMC1169032

[24] Russ CF Yanko WA Factors affecting salmonella’s repopulation in composted sludges Appl. Environ. Microbiol 1981 41 597 602 7224626 10.1128/aem.41.3.597-602.1981 PMC243745

[25] Pflug IJ Holcomb RG Gomez MM Principles of the thermal destruction of microorganisms Disinfection, Sterilization and Preservation5th ed Block SS Lippincott, Williams and Wilkins Philadelphia, PA, USA 2001 79 129

[26] Moats WA Dabbah R Edwards VM Interpretation of nonlogarithmic survivor curves of heated bacteria J. Food Sci 1971 36 523 526

[27] Doyle ME Mazzotta AS Review of studies on the thermal resistance of Salmonellae J. Food Prot 2000 63 779 795 10852574 10.4315/0362-028x-63.6.779

[28] Chiruta J Davey KR Thomas CJ Thermal inactivation kinetics of three vegetative bacteria as influenced by combined temperature and pH in a liquid medium Food Bioprod. Process 1997 75 174 180

[29] Smith MG Survival of *E. coli* and *Salmonella* after chilling and freezing in liquid media J. Food Sci 1995 60 509 512

[30] Spinks AT Dunstan RH Harrison T Coombes P Kuczera G Thermal inactivation of water-borne pathogenic and indicator bacteria at sub-boiling temperatures Water Res 2006 40 1326 1332 16524613 10.1016/j.watres.2006.01.032

[31] Sharma S Sachdeva P Virdi JS Emerging water-borne pathogens Appl. Microbiol. Biotechnol 2003 61 424 428 12684849 10.1007/s00253-003-1302-y

[32] Godfree A Farrell J Processes for managing pathogens J. Environ. Qual 2005 34 105 113 15647539 10.2134/jeq2005.0105

[33] Hay JC Pathogen destruction and biosolids compost Biocycle 1996 37 67 77

[34] United States Environmental Protection Agency; 40 CFR Part 503. Standards for the use or disposal of sewage sludge Fed Regist 1994 58 9248 9415

[35] Finkel SE Kolter R DNA as a nutrient: novel role for bacterial competence gene homologs J. Bacteriol 2001 183 6288 6293 11591672 10.1128/JB.183.21.6288-6293.2001 PMC100116

[36] Nielsen KM Johnsen PJ Bensasson D Daffonchio D Release and persistence of extracellular DNA in the environment Environ .Safety Res 2007 61 37 53 10.1051/ebr:2007031 17961479

[37] Chen I Dubnau D DNA uptake during bacterial transformation Nat. Rev. Microbiol 2004 3 241 249 15083159 10.1038/nrmicro844

[38] Dubnau D DNA uptake in bacteria Annu. Rev. Microbiol 1999 53 217 244 10547691 10.1146/annurev.micro.53.1.217

[39] Weiss MS Abele U Weckesser J Welte W Schiltz E Schulz GE Molecular architecture and electrostatic properties of a bacterial porin Science 1991 254 1627 1630 1721242 10.1126/science.1721242

[40] Paul JH Jeffrey WH Deflaun MF Dynamics of extracellular DNA in the marine environment Appl. Environ. Microbiol 1987 53 170 179 3827244 10.1128/aem.53.1.170-179.1987 PMC203621

[41] Burns R Enzymes in the Environment: Activity, Ecology and Applications CRS Press, Taylor and Francis Group New York, NY, USA 2002

[42] Aardema BW Lorenz MG Krumbein WE Protection of sediment-adsorbed transforming DNA against enzymatic inactivation Appl. Environ. Microbiol 1983 46 417 420 16346365 10.1128/aem.46.2.417-420.1983 PMC239404

[43] Buchanan JT Simpson AJ Aziz RK Liu GY Kristian SA Kotb M Feramisco J Nizet V DN*ase* expression allows the pathogen group a *Streptococcus* to escape killing in neutrophil extracellular traps Curr. Biol 2006 16 396 400 16488874 10.1016/j.cub.2005.12.039

[44] Citak S Varlik O Gundogan N Slime production and DN*ase* activity of Spaphylococci isolated from raw milk J. Food Saf 2007 23 281 288

[45] Kneitel JM Chase JM Trade-offs in community ecology: linking spatial scales and species coexistence Ecol. Lett 2004 7 69 80

[46] Ruiz TR Andrews TR Smith GB Identification and characterization of nuclease activities in anaerobic environmental samples Can. J. Microbiol 2000 46 736 740 10941520

[47] Ni H Bacteria learn antibiotic resistance in the sludge Drug discov.Today 2003 8 10 11 10.1016/s1359-6446(03)02908-8 14690625

[48] Coughter JP Stewart GJ Genetic exchange in the environment Antonie Leeuwenhoek 1989 55 15 22 2662898 10.1007/BF02309615

[49] Redfield RJ Genes for breakfast: The have-your-cake-and-eat-it-too of bacterial transformation J. Hered 1993 84 400 404 8409360 10.1093/oxfordjournals.jhered.a111361

[50] White PA McIver CJ Rawlinson WD Integrons and gene cassettes in the Enterobacteriaceae Antimicrob. Agents Chemother 2001 45 2658 2661 11502548 10.1128/AAC.45.9.2658-2661.2001 PMC90711

[51] Moura A Henriques I Ribeiro R Correia A Prevalence and characterization of integrons from bacteria isolated from a slaughterhouse wastewater treatment plant J. Antimicrob. Chemother 2007 60 1243 1250 17913715 10.1093/jac/dkm340

[52] da Costa PM Vaz-Pires P Bernardo F Antimicrobial resistance in Enterococcus spp. isolates in inflow, effluent and sludge from municipal sewage waste treatment plants Water Res 2006 40 1735 1740 16603222 10.1016/j.watres.2006.02.025

[53] Schwartz T Kohnen W Janses B Detection of antibiotic resistant bacteria and their resistance genes in wastewater, surface water, and drinking water biofilms FEMS Microbiol. Ecol 2003 43 325 335 19719664 10.1111/j.1574-6941.2003.tb01073.x

[54] Tennstedt T Szczepanowski R Braun S Occurrence of integron-associated resistance gene cassettes located on antibiotic resistance plasmids isolated from a wastewater treatment plant FEMS Microbiol. Ecol 2003 45 239 252 19719593 10.1016/S0168-6496(03)00164-8

[55] McPherson P Gealt MA Isolation of indigenous wastewater bacterial strains capable of mobilizing plasmid pBR325 Appl. Environ. Microbiol 1986 51 904 909 3524455 10.1128/aem.51.5.904-909.1986 PMC238985

[56] Lorenz MG Wackernagel W Bacterial gene transfer by natural genetic transformation in the environment Microbiol. Rev 1994 58 563 602 7968924 10.1128/mr.58.3.563-602.1994 PMC372978

[57] Levy SB The challenge of antibiotic resistance Sci. Am 1998 278 32 39 10.1038/scientificamerican0398-46 9487702

[58] Lindberg RH Björklund K Rendahl P Environmental risk assessment of antibiotics in the Swedish environment with emphasis on sewage treatment plants Water Res 2007 41 613 619 17187841 10.1016/j.watres.2006.11.014

[59] Schwarzenlander C Averhoff B Characterization of DNA transport in the thermophilic. bacterium Thermus thermophilus HB27 FEBS 2006 273 4210 4218 10.1111/j.1742-4658.2006.05416.x 16939619

[60] Layden N An evaluation of autothermal thermophilic aerobic digestion (ATAD) of municipal slugde in Ireland J. Environ. Eng. Sci 2007 6 19 29

[61] Frydrych I Dziworska G Bilska J Comparative analysis of the thermal insulation properties of fabrics made of natural and man-made cellulose fibres Fibres Text East Eur 2002 10/12 40 44

[62] Pereira-Neto JT Stentiford EI Smith DV Survival of faecal indicator micro-organisms in refuse/sludge composting using the aerated static pile system Waste Manage. Res 1986 4 397 406

[63] Plym-Forshell L Survival of salmonellas and Ascaris suum eggs in a thermophilic biogas plant Acta veter. Scand 1995 36 79 85 10.1186/BF03547704 PMC8095423 7572460

[64] Shuval H Jodice R Consiglio M Spaggiarri G Spigoni C Control of enteric micro-organisms by aerobic-thermophilic co-composting of wastewater sludge and agro-industry wastes Water Sci Technol 1991 24 401 405

[65] Soares HM Cardenas B Weir D Switzenbaum MS Evaluating pathogen regrowth in biosolids compost BioCycle 1995 36 70 74

[66] Holmes B Willcox WR Lapage SP Identification of Enterobacteriaceae by the API 20E system J. Clin. Pathol 1978 31 22 30 342546 10.1136/jcp.31.1.22 PMC476713

[67] Nubel U Engelen B Felske A Snaidr J Wieshuber A Amann RI Ludwig W Backhaus H Sequence heterogeneities of genes encoding 16S rRNAs in *Paenibacillus polymyxa* detected by temperature gradient gel electrophoresis J. Bacteriol 1996 178 5636 5643 8824607 10.1128/jb.178.19.5636-5643.1996 PMC178401

[68] Fisher MM Triplett EW Automated approaches for ribosomal intergenic spacer analysis of microbial diversity and its application to freshwater bacterial communities Appl. Environ. Microbiol 1999 65 4630 4636 10508099 10.1128/aem.65.10.4630-4636.1999 PMC91617

[69] Nagpal ML Fox KF Fox A Utility of 16S-23S rRNA spacer region methodology: how similar are interspace regions within a genome and between strains for closely related organisms? J. Microbiol. Meth 1998 33 211 219

[70] Yu Z Mohn W Bacterial Diversity and Community Structure in an Aerated Lagoon Revealed by Ribosomal Intergenic Spacer Analyses and 16S Ribosomal DNA Sequencing Appl. Environ. Microbiol 2001 67 1565 1574 11282606 10.1128/AEM.67.4.1565-1574.2001 PMC92770

[71] Scheinert P Krausse R Ullman U Soller R Krupp G Molecular differentiation of bacteria by PCR amplification of the 16S-23S rRNA spacer J. Microbiol. Methods 1996 26 103 117

[72] Sambrook J Fritschi EF Maniatis T Molecular Cloning: A Laboratory Manual Cold Spring Harbor Laboratory Press New York, NY, USA 1989

[73] McGrath BM O’Halloran JA Piterina AV Pembroke JT Molecular tools to detect the IncJ elements: A family of integrating, antibiotic resistant mobile genetic elements J. Microbiol. Methods 2006 66 32 42 16316703 10.1016/j.mimet.2005.10.004

[74] Piterina AV Bartlett J Pembroke JT Molecular Analysis of Bacterial Community DNA in Sludge Undergoing Autothermal Thermophilic Aerobic Digestion (ATAD): Pitfalls and Improved Methodology to Enhance Diversity Recovery Diversity 2010 2 505 526

[75] Sanger F Nicken S Couslon AR DNA sequencing with chain-terminating inhibitors Biotechnol 1992 24 104 108 1422003

[76] Altschul SF Madden TL Schaffer AA Zhang J Zhang Z Miller W Lipman DJ Gapped BLAST and PSI-BLAST: a new generation of protein database search programs Nucleic Acids Res 1997 25 3389 3402 9254694 10.1093/nar/25.17.3389 PMC146917

[77] Stackebrandt E Goebel BM Taxonomic note: a place for DNA-DNA reassociation and 16S rRNA sequence analysis in the present species in bacteriology Int. J. Syst. Bacteriol 1994 44 846 849

[78] Drancourt M Bollet C Carlioz A Martelin R Gayral JP Raoult D 16S ribosomal DNA sequence analysis of a large collection of environmental and clinical unidentifiable bacterial isolates J. Clin. Microbiol 2000 38 3623 3630 11015374 10.1128/jcm.38.10.3623-3630.2000 PMC87447

[79] Randolph MK William J Fate of pathogens in thermophilic aerobic sludge digestion Wat. Res 1982 16 1051 1060

[80] Golueke CG When is compost “safe”? The Art and Science of Composting JG Press, Inc Emmaus, PL, USA 1991 220 229

[81] Dumontet S Dinel H Baloda SB Pathogen reduction in sewage sludge by composting and other biological treatments: A review Biol. Agr. Hortic 1999 16 409 430

[82] Emerson KR Lund RE Thurston RV Aqueous ammonia equilibrium calculations: Effects of pH and temperature J. Fish. Res. Board Can 1975 32 2379 2383

[83] Park GW Diez-Gonzalez F Utilization of carbonate and ammonia based treatments to eliminate *Escherichia coli* O157:H7 and S*almonella* 42 *Typhimurium* DT104 from cattle manure J. Appl. Microbiol 2003 94 675 685 12631203 10.1046/j.1365-2672.2003.01899.x

[84] Mendez JM Jimenez BE Barrios JA Improved alkaline stabilization of municipal wastewater sludge Water Sci. Technol 2002 46 139 146 12479463

[85] Mendez JM Jimenez B Maya C Disinfection kinetics of pathogens in physicochemical sludge treated with ammonia Water Sci. Technol 2004 50 67 74 15580996

[86] Ottoson J Nordin A von Rosen D Vinnerås B Salmonella reduction in manure by the addition of urea and ammonia Bioresource. Technol 2007 99 1610 1615 10.1016/j.biortech.2007.04.009 17531477

